# Formulating a multi-layered framework for automobile architecture

**DOI:** 10.1038/s41598-022-05156-4

**Published:** 2022-01-27

**Authors:** Sushil Chandra

**Affiliations:** BML Munjal University, G-29A, GF, South City-2, Gurgaon, 122018 India

**Keywords:** Aerospace engineering, Mechanical engineering

## Abstract

Product architecture is defined as allocation of components to functions and their interfaces. But what makes this formulation insufficient for automobile architecture is their multi-layered allocation of components and the visual aspect of the architecture. This paper suggests, through an empirical study of two-wheeler models being manufactured by a prominent manufacturer, a multi-tiered framework along with a visual template for two-wheeler architecture which includes their visual schema as well. This paper investigates and demonstrates that this framework and template satisfy the requirements of product architecture by using examples from the domain of motorcycle design. Further, this paper investigates the utility of this framework for platforming strategy, innovation, adoption of new technology and standardization.

## Introduction

Automobile architecture is a concept which has currently gained in significance, especially considering architectural transition due to the onslaught of artificial intelligence and electric mobility. What adds to its significance is the urgent need for a suitable formulation and framework fulfilling the specific needs of automobiles. But the question to be addressed before discussing this suitable formulation for automobile architecture is about the definition of architecture. Though, the word ‘architecture’ has its origin in ancient times, the exact contours of the term have not been clear for a very long time. Now since the usage of the word has widened its scope beyond civil structures to software, manufacturing, automobiles and even arts and literature, it has become even more important to clearly define it so that the contours are clearly identifiable and verifiable. Before ISO/IEC/IEEE (2011)^1^ defined architecture of a system as “fundamental of a system in its environment embodied in its elements, relationships and in the principles of its design and evolution”, Ulrich (1995)^2^ provided a simpler sounding definition for product architecture. It is a scheme by which the function of a product is allocated to physical components. Crawley (2007)^3^ defines architecture similarly as “the embodiment of concept, and the allocation of physical/informational function to elements of form, and definition of interfaces among the elements and with the surrounding context”. Further simplifying it, he defines architecture as “the details of the assignment of function to form, and the definition of interfaces”. Dori (2002)^4^ defines the architecture as “the overall system's structure-behaviour combination, which enables it to attain its functions while embodying the architect's concept”. Rechtin and Maier (2002)^5^ define the architecture of a system as “the structure—in terms of components, connections, and constraints—of a product, process, or element”. So, there is near unanimity on the definition of architecture that it is the allocation of systems or components to functions and interfaces. Here we come across another term ‘system’ that needs a clear and objective definition. Crawley (2007) defines it as “a set of interrelated elements which perform a function, whose functionality is greater than the sum of the parts”. Dori (2007) defines it as “an object that carries out or supports a significant function”. Rechtin and Maier (2002) define it as “a collection of different things, which together produce results unachievable **by** the elements alone”. Going by these definitions, any product (or, in our case automobile) itself is a system which is a set of systems performing their respective functions.

Thus, we get a clear and objective definition of both ‘architecture’ and ‘system’ but the questions that remain unanswered are:

1. What are the unique aspects that characterize automobile architecture?
2. How do we define a ‘layer’ or ‘level’ in context of automobile architecture?
3. How do we define automobile architecture with respect to the layers of architecture?

Looking for the answer to the first question regarding the uniqueness of automobile architecture, Ristic (1988)^6^ provides a probable answer where he lists the following unique aspects of automobile architecture:

- Balance of various functions by managing the contradictory demands of functions: Though this applies to various other architectures, this becomes critical for automobiles because the absence of this contradiction management will render the automobile dysfunctional.
- Managing the contradiction between symmetry and asymmetry.
- Anti-perspective three-dimensionality: The element of space in time is generated due to the unique factor of rectilinear motion, where the perspective view becomes irrelevant due the constantly changing viewpoint. Moreover, the law of hydrodynamics makes the three-dimensional form very critical so that it penetrates air and water with least resistance. Both, the factors make the form and the motion dialectically dependent on each other.

Besides these unique characteristics, one unique feature of automobile architecture is the intricate and interactive interfacing of the user’s body with the product. This unique aspect is not applicable in any other architecture. (Yes, of course, there are other products where user’s body interfaces with the product, but these interfaces don’t form the central narrative of the architecture, the way they form in case of automobiles). Interspersing this most unique aspect of form-motion-user’s body interaction into the general definition and framework of architecture, this interaction becomes an essential function to which systems and components must be assigned, after this interaction is translated into more concrete and identifiable elements.

With a simple characterization for vehicle architecture thus available, we must consider whether it fulfils the need. Definitions and frameworks are useful only if we can put the definitions to practical use. And, for this we need a framework which describes the architecture of an automobile objectively, crisply and in simple words. In fact, Ulrich (1995) has already put in place a comprehensive framework for product architecture. Let us have a brief look at this framework. Since he defines product architecture as allocation of physical components to functions, the framework has been derived from this perspective. He defines product architecture as (1) the arrangement of functional elements (2) the mapping of functional elements (the allocation of elements to functions) and (3) the specification of interfaces among physical components. This results in a typology where architecture is classified as integral and modular and interfaces between elements are typified as coupled and decoupled on one hand and slot, bus, and sectional on the other. The fundamental issue with this framework is that this is redundant in context of vehicle architectures. In general context of industrial or consumer products this provides a good foundation to describe and classify the architecture but in context of vehicle design, the design of all vehicles is modular in architecture and the interfaces in all designs are slot interface. Toepfer and Naumann (2016)^7^ have described vehicle architecture as something which “describes the physical layout of a vehicle and the way it realizes its function by a given set of basic architectures parameters and modules”. They, thus, suggest a method to develop a vehicle architecture in a CAD-PLM-database framework. But what makes the description of vehicle architecture difficult is its complex (hundreds of elements interconnected to each other through innumerable interfaces) and multi-layered structure. Cameron et al. (2016)^8^ have defined the levels of system architecture and provided methods to describe the architectures, but they are fraught with two issues—(a) The methods they have described (SysML and OPM) are not layer specific and (2) their definition of layers and their identification does not help in clearly separating the layers while describing them. Chuma (2006)^9^, in his study on system complexity and product organization, states that with rapidly increasing complexity, new organizational forms become inevitable So, with huge variation in architecture of vehicles and with ever-increasing complexity of automobile design, a new framework for architecture becomes inevitable, which can address this complexity, the visual aspect and the issue of layers or levels.

With these unique characteristics of automobile architecture and considering the quest for a framework incorporating them with concrete and identifiable elements, when we look for the layered frameworks for product architecture, we get two formulations. The first is by Kinnunen (2006)^10^, who provides a three level (Level 0 combining needs, concept, processes and objects, level 1 combining specializing intents, zooming processes, and decomposing objects and level 2 combining decomposing and zooming objects and processes) model using Object-Process Methodology (OPM). The second framework is provided by Cameron et al. (2016) which also uses the Object-Process Methodology but with the difference that here the number of levels is not limited, and the models are common for all levels.

Both these frameworks can be applied to automobiles, but the unique characteristics of automobile architecture will remain unaddressed. The second issue is that these models are too complicated and mathematical to be comprehended in one glance. For example, for two-wheelers, the words ‘motorcycle’ and ‘scooter’ combine the visual schema, the engineering schema, and the man–machine interface to create motion in one single word which no mathematical formulation can achieve. So, this becomes the top-level of architecture where the semantics, the engineering, and the form-function-emotion triad merge in simple sounding words. This can be either named super-architecture or simply product-architecture. The second level (macro-level or system-level) is one where the automobile is expressed as a super-system combining many systems assigned to separately identifiable functions through interfaces. The pre-requisite again is objectivity (where every architecture is uniquely expressed) and simplicity such that it can easily be translated into an algorithm. The third level is component/ sub-system level or micro-level where a component or sub-system is broken into individual unbreakable parts and the architecture is expressed as their assignment to individual micro-functions and interfaces.

## Objective

The formulation by Ulrich provides a good foundation to describe architecture in general and it applies to architecture of automobiles as well. Still, what needs further investigation is the level or zooming in and zooming out view of architecture. What it means is that the allocation of functions to components and defining the interfaces in an automobile is a multi-layered exercise and architectural decisions are to be taken differently at different layers. The architectural decisions, while choosing the broader aspects of design, are completely different from the ones at the microscopic level. The objective of this paper is to go beyond Ulrich’s formulation and

- Define the architecture of an automobile at different layers of design.
- Create objective formulations for the architectures at the various layers defined in the first step in a way that the description of architecture becomes unique.
- Create a formulation that considers the visual aspect of automobile design.

## Justification

**Figure 1:**
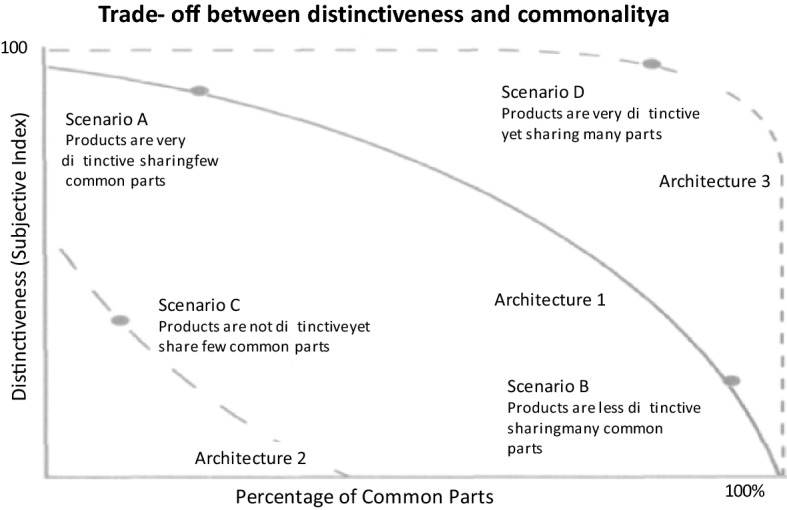
Trade-off between differentiation and commonality (reference: Ulrich).

An obvious question that needs to be answered before starting this exercise is that whether this exercise is really needed and if the answer is in affirmative, what purposes will be served by this exercise. Let us start by asking, what exactly is the problem with the formulation provided by Cameron et al. (2016) where they have described two methods described as SysML and OPM. We will see that the processes that can be conducted with the proposed formulation to achieve the objectives described in the following paragraphs cannot be achieved with these formulations. The following factors make it imperative that automobile architecture is defined and formulated in an objective and nuanced way which addresses various levels of automobile design (which SysML and OPM do not):

- Platform strategy: Ulrich (1998)^11^, has established a relationship between architecture and the extent of commonality. Now, this commonality itself can be at many levels (i.e., at the level of components, assemblies, or systems) and therefore, the architecture must be defined level-wise. Not only this, any change in extent of commonality affects the components allocated to functions and their interfaces. Inversely, any change in components allocated to functions and their interfaces affects the level of commonality (Ref Fig. 1).
- Facilitating a technology change: Three factor are constantly forcing automobile manufacturers to constantly upgrade the technology: (a) Approval standards for environment (b) Alternatives to fossil fuels and (c) Digitalization. This makes it essential for designers to constantly change (a) the various functions available in an automobile (b) Components allocated to these functions and (c) the interfaces between the changed components.

Now, problems arise because of the location of these changes in the hierarchy of components. Let us consider for example, the addition of catalytic converters to the silencer of a motorcycle.

This necessitates the addition of a new function (oxidation of carbon monoxide in exhaust gases), allocation of a new component (catalytic converter) and the change in the interface with the design of silencer. But these changes occur at the tertiary level of design without affecting the motorcycle architecture at the product level or system level. But if the manufacturer wants to make an electric vehicle, a completely new architecture is required, which does not only change the visual schema of a motorcycle but the overall list of functions with various additions (like battery management), completely new components at primary level (like motor, battery etc.) and new interfaces. This means not all technological evolutions change the architecture at same level. This makes it is essential to define and formulate automobile architecture in a multi-level way and taking the visual schema into account.

**Figure 2:**
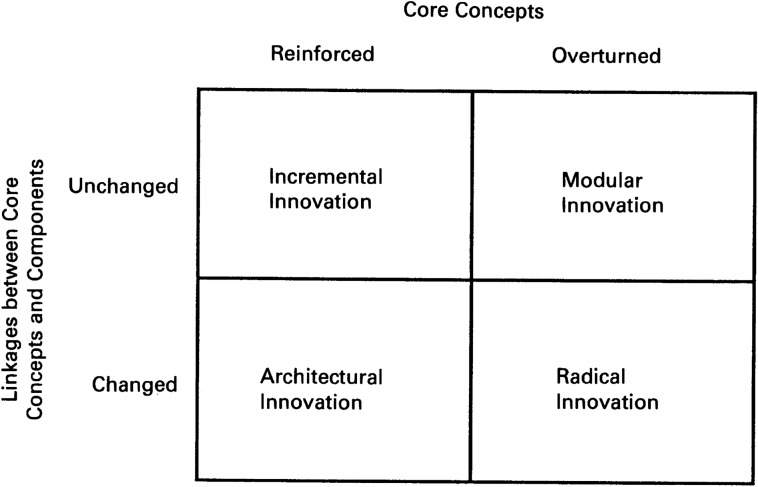
Innovation quadrant (reference: Henderson and Clark).

(c) Innovation: Henderson and Clark (1990)^12^ have formulated an innovation quadrant (refer Fig. 2), which clearly differentiates between different types of innovation based on the combination of interfaces and technology.

What this formulation clearly highlights is that for any innovation to take place, it is essential to clearly identify the interfaces (or linkages) and core concepts. Now, any complex system, and specially automobiles, consist of these linkages and core concepts at various levels But, what is common in all cases is that it needs, at least in many cases, a change in architecture, as by definition even radical innovation needs a change in architecture. And, since it happens at many levels, a multi-level approach to define and formulate architecture is essential.

(d) Standardization: Standardization is the broadest level of commonization, which generally happens at the tertiary level of design. Commonization generally happens across models of a manufacturer, whereas standardization happens across manufacturers across the globe. It happens not only with components (bolts, batteries, tires etc.) but with design features as well (e.g., plugs and holes dimensions) and even material compositions. Now, a tiered description of architecture helps in identifying the components and interfaces where differentiation is not important enough to warrant a new design (battery) and where a global cooperation is essential (material compositions, plug and hole dimensions).

## Methodology

The major source of the study was design data related to a genre of automobiles encompassing as many variations of design as possible. The basic approach was to try and create a formulation which covers all these variations of design. Since the area of expertise of the author is motorcycle and scooter design, the genre selected was two-wheeler design. The steps we will follow are as follows:

- We will create a layer-wise definition of automobile architecture and a formulation which helps in objectively describing architecture at different layers of design.
- We will validate our formulation to check whether it is valid across all possible designs at all levels.
- We will further validate to check whether it fulfils all our stated objectives as stated in our justification.

## Literature review

Jiao et al. (2001)^13^ define the architecture of product family in terms of three elements-common base, differentiation enabler and configuration mechanism. This is a unique formulation, and it indisputably helps in devising platform strategy. But our challenge is to define the architecture of a product in such a way that it addresses the multi-layered structure of an automobile. Moreover, Jiao’s model does not account for visual elements and their interfacing. Ristic (1988) provides a parallel with building architecture which includes visual architecture as well, but it does not provide adequate basis for domain modeling and therefore, an objective formulation. Chandra (2015)^14^ also provides a formulation which considers visual schema specifically, but specific to the context of motorcycles only and without considering the multi-tiered structure. Pelliccione et al. (2017)^15^ have devised an architectural framework for system architecture of cars but only for software development. The common point with all these formulations is that they do not offer a standpoint from where we can explore a formulation for multi-tiered structure of automobiles. This is where, the formulation by Ulrich (1995) proves to be the most suitable starting point because he defines product architecture as (1) the arrangement of functional elements (2) the mapping of functional elements and (3) the specification of interfaces among physical components. This definition applies to automobile architecture suitably except for the fact that visual architecture is not accounted for as an independent criterion, and it does not cater to multi-tiered structures.

## Discussion

An automobile is a multi-layered structure. A cursory look at the bill of material of any automobile-from the simplest motorized bicycle to the most complicated limousine-will testify to this. But, broadly speaking we can specify three tiers. The very first look at an automobile gives a birds-eye view, and this look gives a lot of architectural information-both visual and technological. We propose to call the summation of this information as super architecture or product architecture. Drilling down to the next level, the most pertinent observation is that any automobile is a combination of various systems-each responsible for a broad function- and all systems interfacing with each other. The set of architectural information pertaining to this combination of systems can be called macro-architecture or system-level architecture. Drilling further down, each system consists of various components and each component again being a set of components. The series of layer beneath a layer continues up to the level of every individual component. The architecture of this complicated web can be called micro-architecture or component-level architecture.

- Super-architecture (Product-level architecture):

Since architecture must be expressed in terms of functions, components allocated to these functions and their interfaces, we must consider the over-arching functions which dictate the design of an automobile. Here, for purpose of convenience the genre of two-wheelers has been considered (though we can apply the same logic and sequence to the world of four-wheeler as well). Now, if we take the bill of materials of any scooter or a motorcycle and write down their primary functions, these functions can be classified under three broad heads:

-Provide mobility to the vehicle.
-Provide access, seating and means of navigation to the user or users.
-Provide a visual and emotional connect to users and those who look at it.

**Figure 3:**
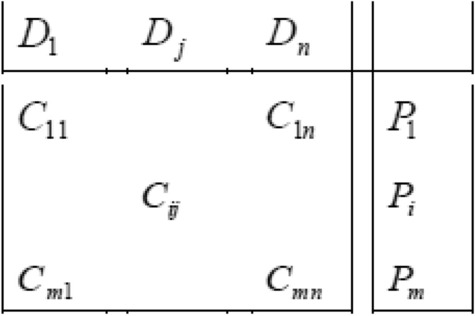
Subjective objective system for generating optimal product concepts (reference: Ziv-Av and Reich).

Before we explore a framework for super architecture for an automobile, it is imperative to look for already available frameworks. One such framework is provided by Ziv-Av and Reich (2003)^16^ in form of subjective-objective systems for generating optimal product concepts. They propose a matrix where the two axes of the matrix are the functions and systems as the diagram below (Fig. 3) shows:

We can see that this formulation, which is fundamentally meant for quantitative evaluation of concepts, does not suit our purpose for two reasons: (a) It does not differentiate between overarching functions and secondary functions and (b) It does not account for interfaces.

Now, we can easily realize that each of these functions has innumerable variety and combinations of designs which can be allocated to them. Our challenge is to put them in manageable number of categories. Let us consider each one of them. As we know, mobility of a vehicle is combination of many basic functions and the components allocated to them are shown in Table 1.

**Table 1:** Components allocated to functions for two-wheelers.

Function	Components		
Power source	Petrol engine	Diesel engine	Electric battery
Conversion to rotary motion	Crank shaft	Crank shaft	Motor
Transmission	Gears, chain, belt	Gears, chain, belt	Gears, chain, belt
Engagement and disengagement	Clutch	Clutch	Switch
Speed variation	Gears, variometric drive	Gears, variometric drive	Electric and electronic devices
Traction	Wheels	Wheels	Wheels

This is only an indicative list, as many auxiliary functions like fuel storage, air intake, exhaust noise reduction have been omitted. But what is important to note from the list is that the set of components can be classified in two broad groups-IC engines (E_1_) and electric engines (E_2_).

**Figure 4:**
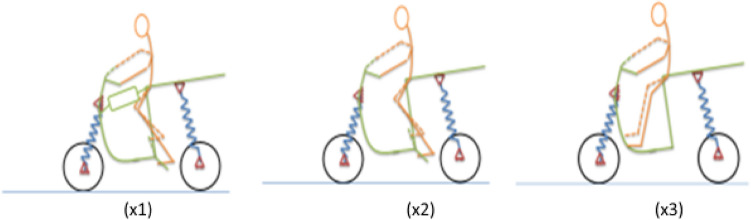
Broad two-wheeler classification based on access.

So far as the function of human access, seating and navigation is concerned, the most important consideration which affects the overall schema is whether the access for the rider is by stepping through the front or by stepping over the seat. This also decides the resting place for the legs of the rider. In case of step-through vehicle, there is a choice available between the legs resting in front or in sides and in case of step-over vehicles, the legs must rest in sides. Another consequence of this classification is the location of fuel tanks. In case of step-through vehicles, the fuel tanks must be located below the seat and the step-over vehicle have their fuel tanks right in front. This classification gets illustrated Fig. 4.

**Figure 5:**
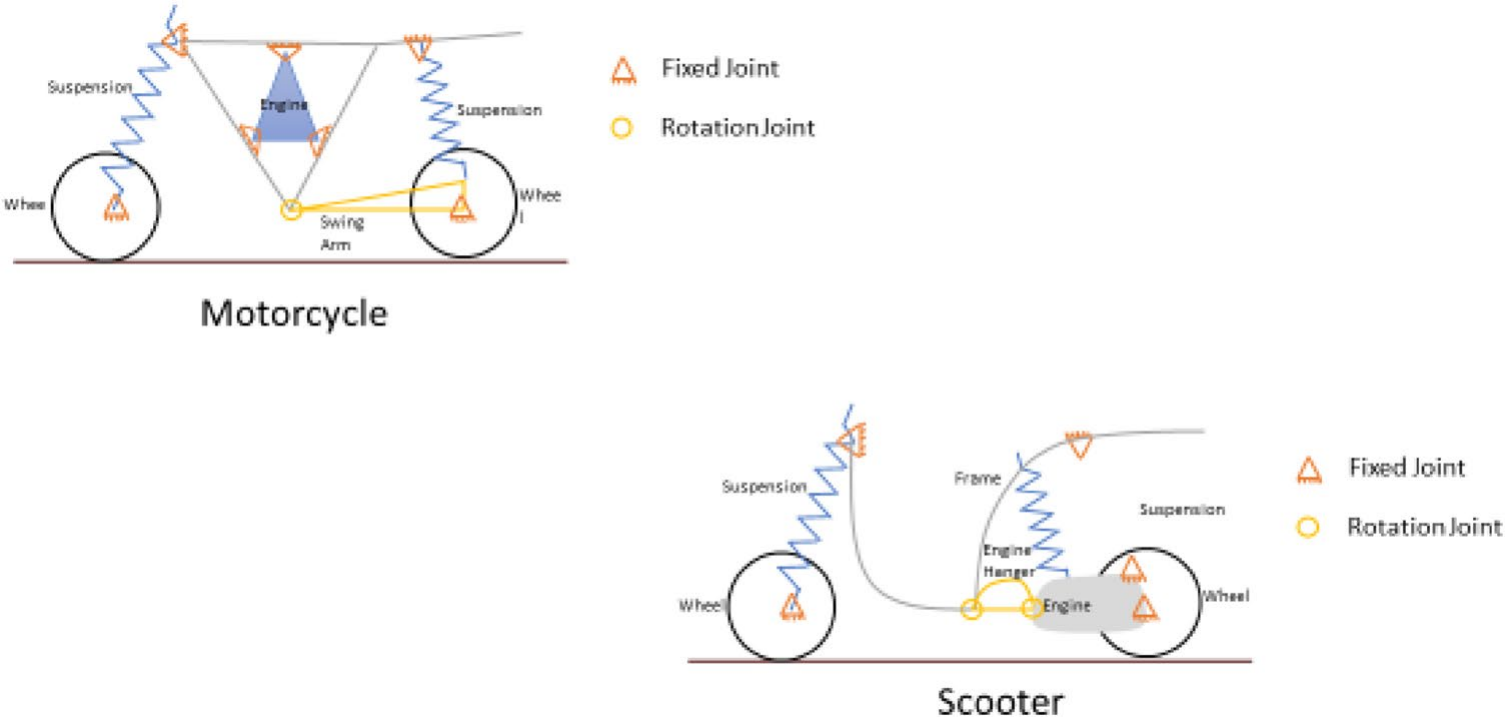
The difference between a motorcycle and a scooter: a schematic diagram.

Beyond this access to the rider and the rider’s posture, which define the fundamental classification of a two-wheeler, there is another fundamental difference which splits the world of two-wheeler vertically-the navigation mechanism which gives the two-wheeler its name i.e., a motorcycle or a scooter. As the diagram shows the motorcycle has an engine rigidly fixed to the frame and the drive transmitted to the rear wheel fixed to a swinging arm normally through a chain and sprocket mechanism. On the other hand, a scooter has the rear wheel fixed directly to the engine, which itself is acting as a swinging arm with respect to the frame (Ref Fig. 5).

What we observe in the last two classifications is a common ground. One can see with quite a good clarity, with the data available with us (and our commonsense logic also matches with it) that the third category in the access and posture classification is exclusive for scooters. Similarly, the first and second are exclusive for motorcycles. So, these two classifications can be easily merged to create three categories—(a) Step-over motorcycles (b) Step-through motorcycles and (c) scooters. By choosing one of the three we distinctly define the access, the posture, and the navigation mechanism.

**Figure 6:**
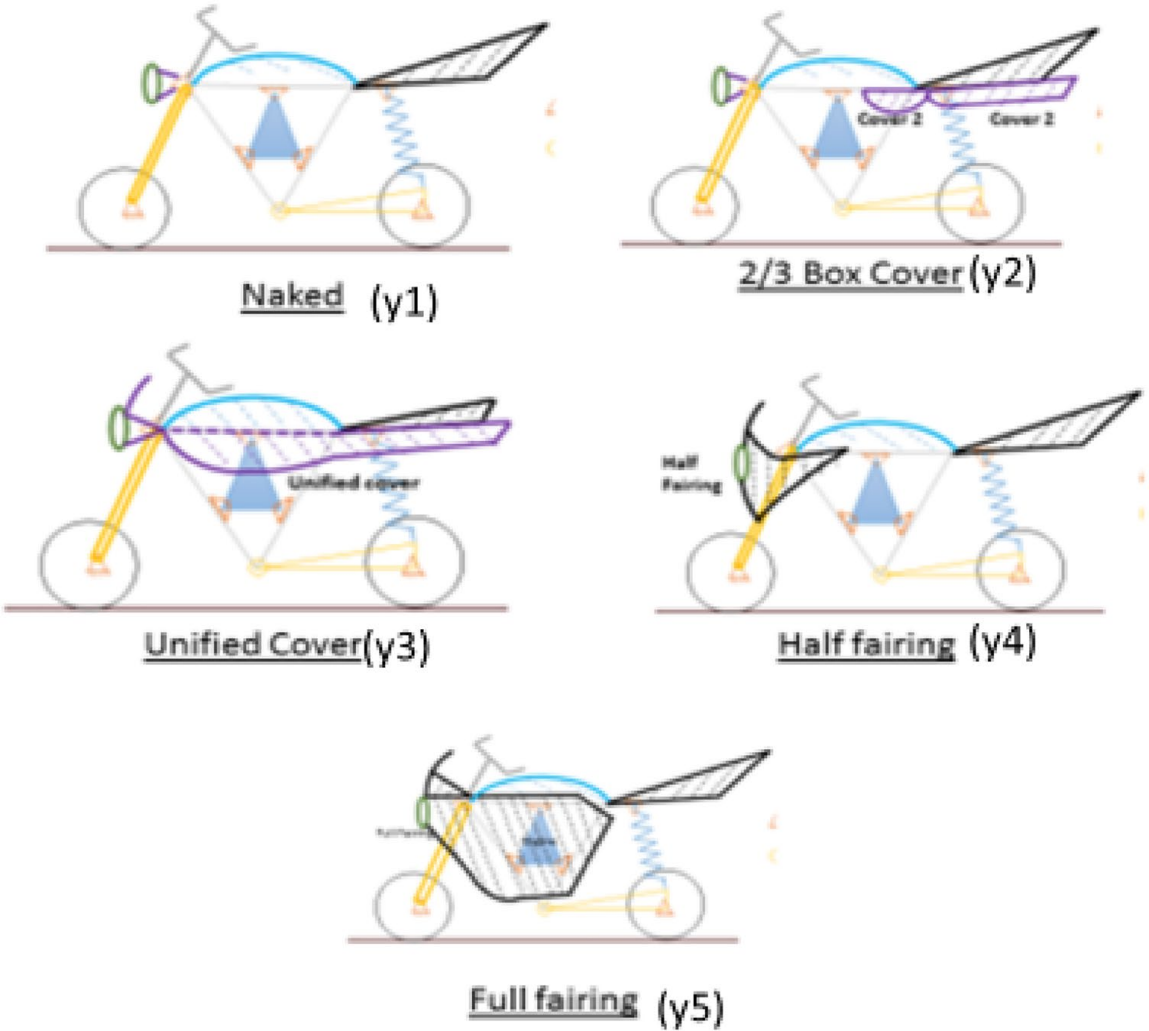
Classification of two-wheelers based on visual schema.

Providing a visual and emotional connect to users and onlookers is a complex process but finally it depends on the visual schema. At broader level, this schema, based on the data available can be classified in categories shown in the following diagram (Ref Fig. 6):

Though one can be sure that this is not an exhaustive list and styling being a creative area, new visual schema is bound to crop up, this classification suffices for our purpose, which is not to provide an exhaustive list. The important point here is that by choosing these three categories—E (E_1_ or E_2_), X (X_1_, X_2_ or X_3_) and Y (Y_1_, Y_2_, ––– or Y_5_), we completely define the broader contours of the architecture. For example, by specifying a two-wheeler as a full fairing step-over, petrol-engine motorcycle, we completely define the super-architecture.

Now, we test this definition by verifying whether it satisfies Ulrich’s (1995) definition of architecture. We can verify by testing both the versions. The first version is easy to verify i.e., whether this definition allocates component to functions and the answer is yes. The second version of the definition (i.e., whether this definition provides the three details—function, the construction of elements and the interfaces) is tricky. So far as the functions and the construction of elements is concerned, they are clearly available. For interfaces, we must look deeper. All the three classifications i.e., motorcycle vs scooter, access (X_1_, X_2_ etc.) and visual schema (Y_1_, Y_2_ etc.) define the interfaces. The classification of motorcycle vs scooter defines the interface between engine, frame, and wheels. The step—through versus step-over classification defines the interface between seat, fuel tank and step-rests. Finally, the visual schema is nothing but the interface between the visual elements. So, our definition passes the scrutiny.

(b) System Level Architecture

What does not emerge from the super-architectural details is the system-level information. For this we drill down a level deeper i.e., macro-architecture or system-level architecture.

The system-level architecture for automobiles can be described through a diagram, which I propose to be called *house of architecture* as it looks very similar in appearance to the house of quality. This diagram divides the major functions into system-level functions like power generation, transmission, shock absorption etc. and then allocates system-level construction units like power-source, transmission, front suspension, rear suspension, front-brake, rear brake, front wheel, rear wheel and front frame, mainframe, rear body, and side body. Then it qualifies the construction classification. It can be easily noted that many functions like fuel supply and seating have been omitted here. This has been done to (a) to reduce the architecture to the bare bones to describe the engineering of a motorcycle and (b) to simplify the definition to avoid making it too wieldy. The top portion of this house of architecture describes the interfaces by way of the type of interfaces (coupled or decoupled), the parts (if any) constructing these interfaces and the number of parts constituting these interfaces. Not only this, in case there are no interface parts, the relationship between the systems interfacing is also mentioned. This house is completed by mentioning the main parts (excluding fasteners, brackets, wires etc.) against each construction unit. Finally, the complete system-level architecture can be described as shown in Table 2.

**Table 2:**
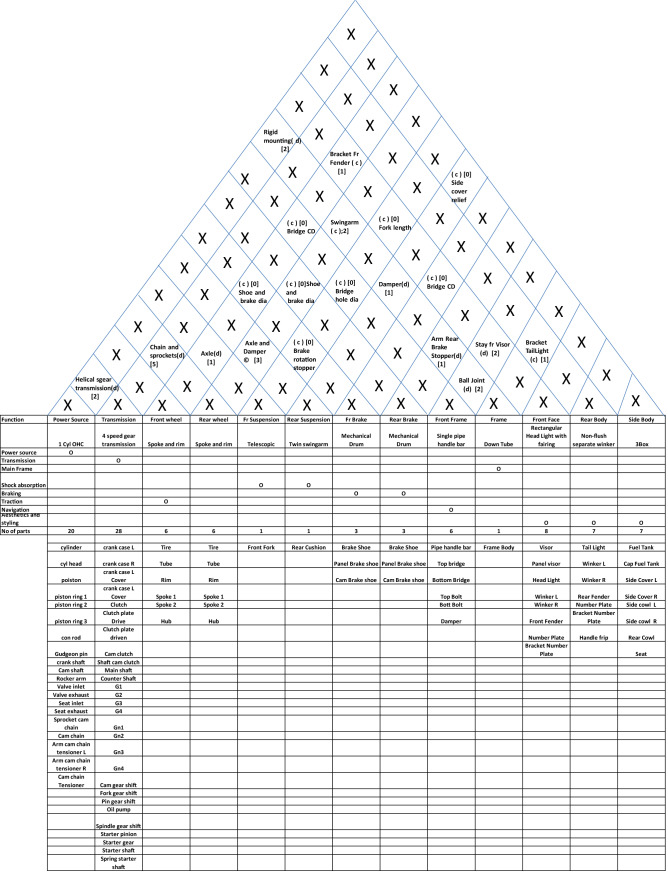
House of architecture for a motorcycle.

Two things can be easily noted here. Nowhere does this architecture describe the quantitative specification. This is important because quantitative specification changes like capacity from 125 to 150 cc or 4 gear transmission to 5 gear transmission does not change the architectural description. Let us argue that there may be cases where quantitative specification changes change the fundamental relationship between the systems. But then, in that case the interface description will change and so we will have a different architecture in any case. The second point is the extension of the same argument that any change in this description fundamentally changes not only the construction classification but in most cases the interfaces as well. For example, if you change the description of power source from 4 stroke overhead cam to V-twin push-rod cam, most probably the interface with frame will change from twin rigid mounting to damped mounting.

Now, let us examine, whether this description satisfies the definition of architecture. The first definition of allocation of components to functions is easily satisfied. The second definition of providing function, construction and interfaces is also easily satisfied. We can put another test to this description—is it possible that two designs having same description may have different architecture. Going by the framework provided here, it is possible that two motorcycles having the same description have completely different looks and styles. But as we have discussed earlier, this framework is for fundamental engineering considerations. If we want to include bodywork and styling as functions, we can easily expand the description framework.

(c) Micro Architecture

The third and the bottom-most layer deals with the component level construction. Frankly, description of the whole micro-architecture of an automobile will be too huge to be accommodated in one description. So, the micro architecture must be described with reference to a limited sub-system (for example, fuel cap or catalytic converters). Having considered this limitation, the framework fundamentally remains the same as that for system-level architecture but with one small difference. This difference is caused by complex mapping of functions to components. While considering system-level architecture, barring some exceptions, it is one to one mapping of functions as each system is supposed to deliver a specific function. But, in case of micro level design such clear segregation is difficult and unnecessary. Keeping this in view, we must provide for complex allocation of components to functions.

**Figure 7:**
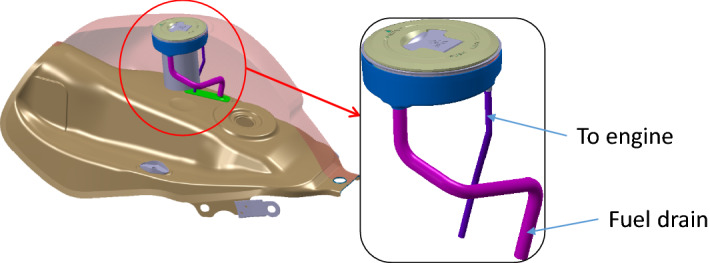
Construction of motorcycle fuel cap.

Let us take an example from real life. Motorcycle fuel tanks have fuel caps fitted into them. In earlier simpler times they had a simple function to enable fuel filling, locking the tank and allowing the gases to escape. Now with regulations to limit the evaporative emissions, these escaping gases must be routed to the engine. So, we see a metallic tube connecting the cap to the engine (Ref Fig. 7).

**Figure 8:**
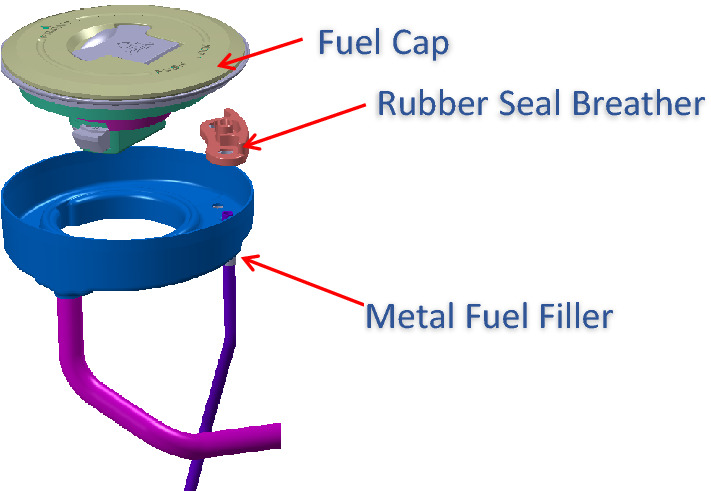
The components of a fuel cap.

So, we have three functions and three components (Ref Fig. 8) here. The three functions, the system is supposed to deliver are (1) to direct all the escaping petrol vapor to canister (which, in turn will redirect it to engine to avoid the vapor escaping to atmosphere) (2) to prevent leakage of vapor and (3) to ensure easy and effective locking of cap. The three components as shown in the diagram are (1) fuel cap (2) rubber seal breather and (3) metal fuel filler.

**Figure 9:**
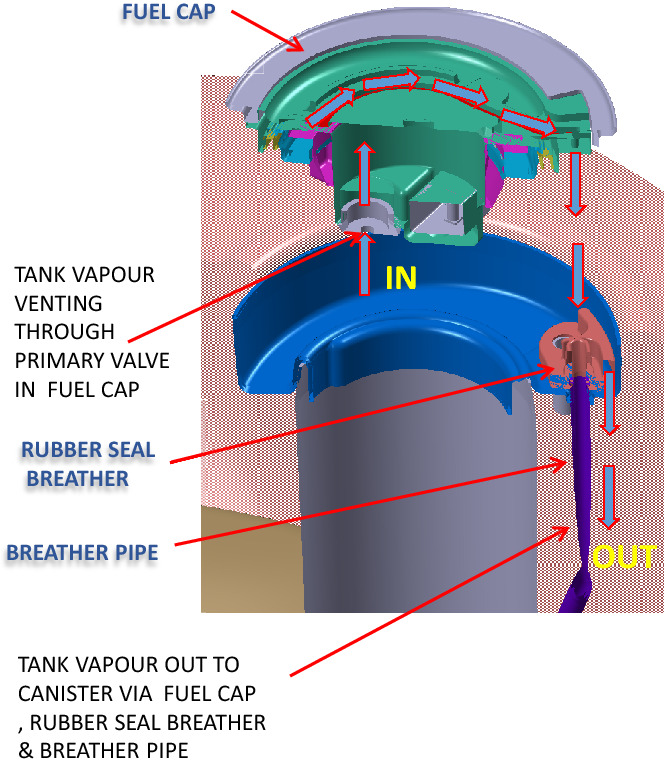
Working of a fuel cap.

So, here we have a metal fuel filler welded on top of the tank, in which a cap is inserted and there is a rubber seal breather between these two parts. To understand the working, we look at the route of passage for the vapor as shown in Fig. 9.

The vapor enters the cap and exits the cap to enter passage in the rubber seal, which is made leak-proof due to the cap pressing against it. The passage has a collar, which is part of the tube carrying the vapor to the engine. (In fact, the vapor is stored in a canister and sucked by the engine when it operates. Now using our architectural framework, the architecture can be described as shown in Table 3.

**Table 3:**
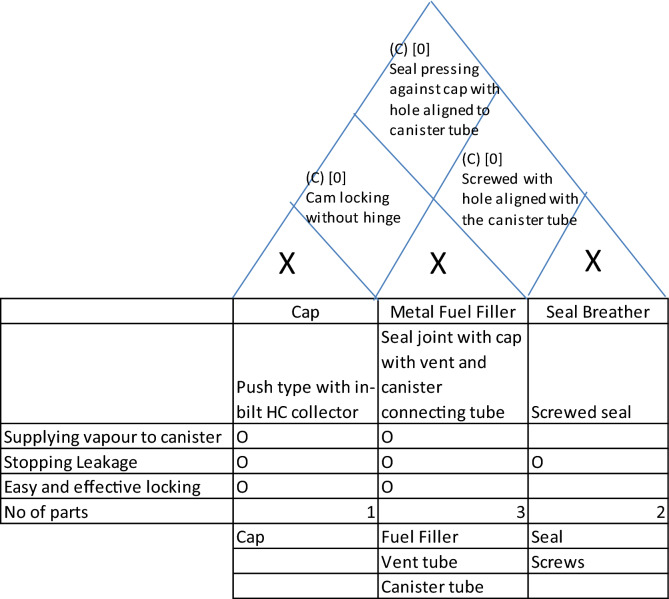
House of architecture for the fuel cap.

Again, examining this framework against the criterions, we find it satisfying them. Now examining the three layers of frameworks, we observe (Ref Table 4) that all of them have some unique characteristics and that is one of the important reasons why it is essential to define them separately.

**Table 4:** Unique characteristics of multi-tiered architecture.

Level	Unique characteristic
Susper architecture	Components and interfaces intermixing with each other
System-level architecture	One to one allocation of components to functions
Micro architecture	Complex allocation of components to functions

## Verification of benefits

The next level of scrutiny for this framework is to examine whether this framework will be suitable for all designs and situations. Let us consider a scenario where either the technology is completely changed (for example electric mobility or autonomous vehicles) or the visual schema is completely unheard of, or the engine frame interface is such that it forms a new category altogether or we can have a situation where all the three possibilities exist together. In all situations the super architecture framework is still valid, and the new architecture can still be described by creating categories for new technology, new visual schema, or new engine-frame interface. So far as system-level architecture is concerned, the framework is so open that any new technology or schema can be accommodated. In system level architecture, old functions can be deleted, and new ones can be created without a hindrance and micro-architecture is, in any case so open that that any mechanism can be explained through it. We will explain all these hypotheses with examples later, while discussing innovation.

The next level of validation for this formulation is to check whether this new formulation is able to meet all our stated objectives. To recap, these objectives are—(a) platforming strategy (b) facilitating new technology (c) innovation (d) standardization.

### Platforming

Ulrich has described the steps for deciding the platforming strategy as-

1. Product plan
2. Differentiation plan
3. Commonality plan
4. Relationship between differentiation and commonality chunks
5. Reconciling the contradictions.

What we are concerned is about the way the architectural framework can help in following these steps. Here we will take a test case from a motorcycle manufacturing and demonstrate how this framework helps in following the steps for platforming strategy. This company at that time was manufacturing three models of motorcycles with super-architecture as follows (Ref Table 5).

**Table 5:** Super-architectures of the three models.

Model	Super-architecture
A	3 Box step-over motorcycle
B	Naked step-over motorcycle
C	3 Box step-through motorcycle

**Figure 10:**
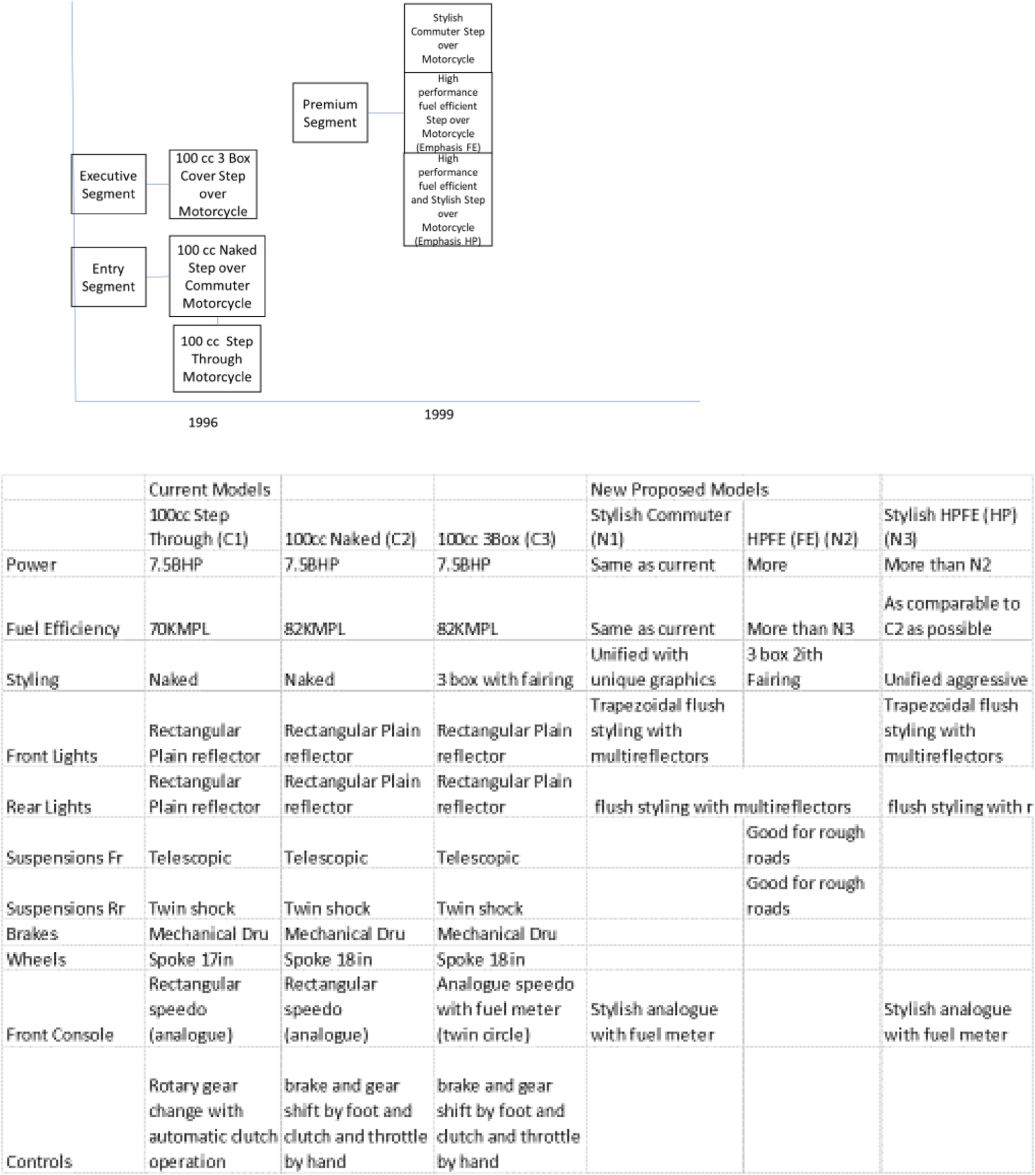
Product plan and differentiation plan.

Out of these three, we have already decsribed the system level architecture for model A. The remaining two have single-cylinder 100 cc OHC engines and backbone frame for model B and monocoque frame for model C. With these three models in the portfolio, the company planned to launch three new models with the following product plan and differentiation plan (ref Fig. 10).

The major challenge after the differentiation plan is to prepare the commonality plan. This needs complete information about the current and new design options. This is where the architectural framework helps as it provides a birds-eye view of the design of new products. So, based on the product plan, we decide the super-architecture as shown in Table 6.

**Table 6:** Super-Architecture of New Models.

	E	X	Y
Stylish commuter step over motorcycle	E_1_	X_1_	Y_3_
High performance fuel efficient step over motorcycle (emphasis FE)	E_1_	X_1_	Y_4_
High performance fuel efficient and stylish step over motorcycle (emphasis HP)	E_1_	X_1_	Y_3_

With this super-architecture in place, the system level architecture creation depends on the following questions:

- Which of the current products can provide the base architecture for the proposed products or can more than one current product do the same?
- Which of the systems are affected by differentiations?
- Whether the affected systems need a change in architecture or only a quantitative change will suffice?
- Whether new functions necessitating new systems need to be added?
- Whether the affected system has a coupled interface with an unaffected system? In that case, whether a change in interface design will be sufficient or the unaffected system needs to change its design or architecture?

Based on these considerations, the base architecture for the entry level unified step-over motorcycle is chosen to be that of model A. Now, answering the five questions, the differential house of architecture with respect to model is drawn, where only the quantity of new parts is shown. Whereever in the interface section the quantity of new parts is shown as zero, it means that the the type and the interface parts remain same as the base product. Another small assumption is that the addition of small brackets in big parts is ignored. This is because the basic rule of automobile platforms is that the same chassis or frames are used in various model by changing the brackets and this is how chassis platforms are generated. So, the differential house of architecture for the stylish entry level bike is shown in Table 7.

**Table 7:**
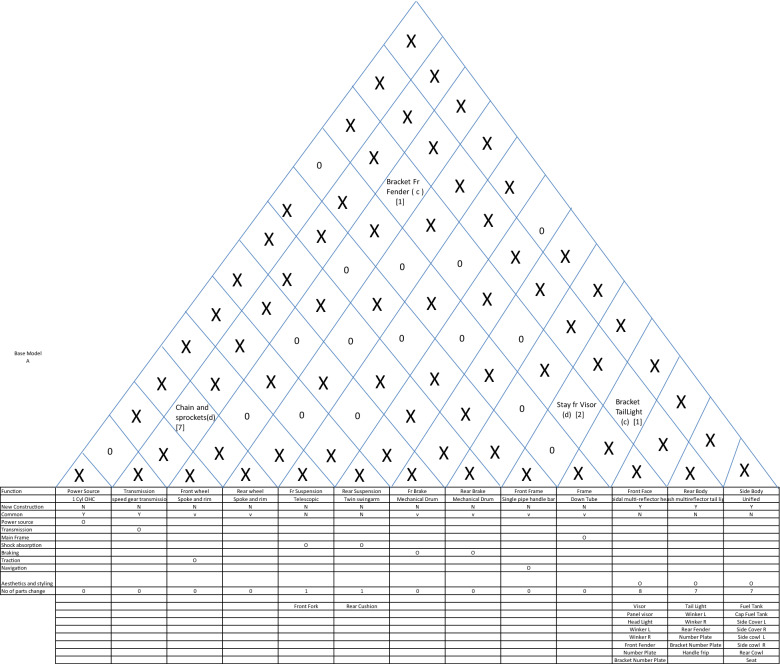
System-level Architecture for Stylish Commuter Bike.

We can very clearly notice here that except for five systems, all other systems are common to the base. Once this house of architecture is devised for a new model, drawing commonality plan and differentiation and commonality chunk relationship becomes very easy. Similarly, the differential houses of architecture for the remaining two new models (high performance and the stylish high-performance fuel-efficient bikes) are shown in Tables 8 and 9 respectively.

**Table 8:**
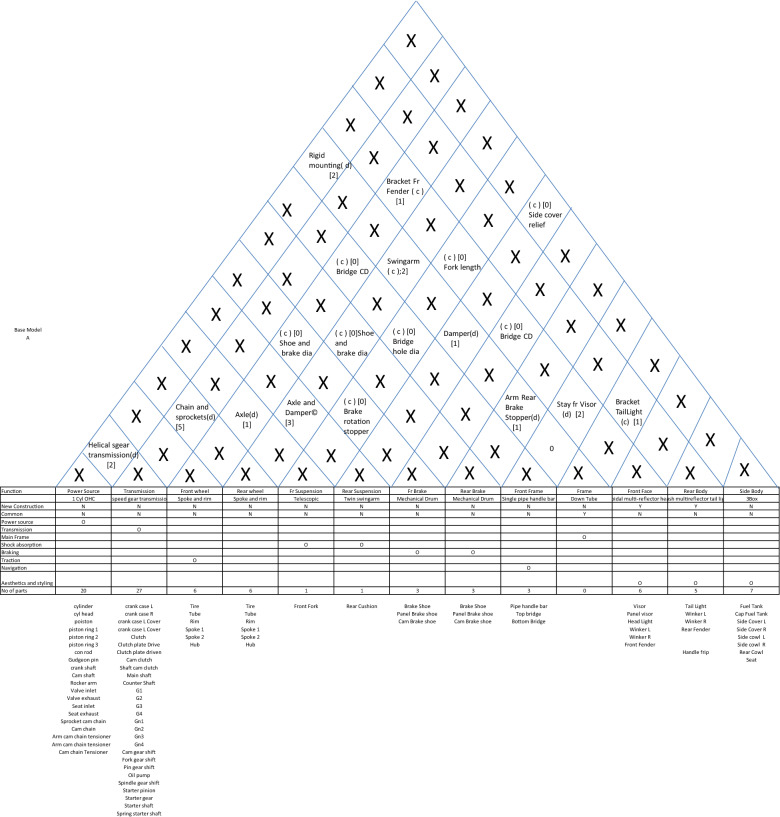
System-level Architecture for High-performance Bike.

**Table 9:**
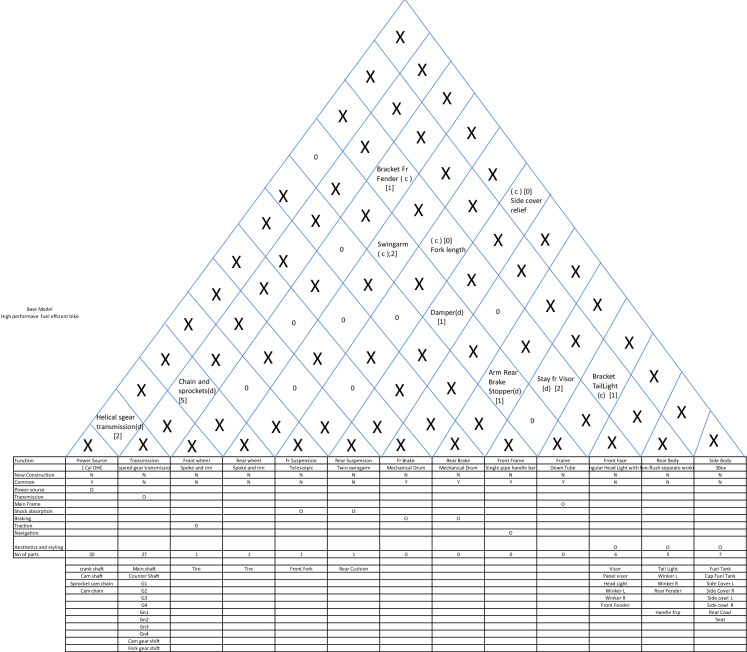
System-level architecture for stylish high-performance bike.

To conclude, all three new proposed products can have the same basic frame as that of model A. One of these three products (the stylish entry level) can have the same engine as well, with only the cushions, lightings and body styling changed to create a new product. The remaining two will have the same basic engine with higher capacity (with some changes in valve timings and transmissions to generate the differentiation between high performance and fuel efficiency). After this analysis, we are in a position where the commonality plan (where the exact number of new and legacy parts and their implications on cost and investment is required) can be easily drawn and the planner can immediately find out where does the integral architecture need to be changed into modular to generate more commonality.

To be precise, this architecture framework has helped in devising the platforming strategy in three ways:

- It has provided the view at a glance so that differentiations and commonalities can be applied with an all-round view.
- It has provided the details of interfaces so that the impact of change in one system on other systems can be visualized.
- It indicates the scope for improving commonality by converting coupled interfaces into decoupled ones.

### Innovation

Referring to the innovation quadrant by Henderson and Clark and the framework, we can link the innovation categories in automobiles to the levels of architecture as follows.

Incremental innovation, in most cases is out of the scope of architectural planning as neither the functional mapping nor the interfaces change at any level. Architectural changes happen mostly at super architectural level because it is at this level where a new product is created without changing the technologies and simply by changing the linkages. Radical innovation happens mostly at macro architectural level because both the core concepts and the linkages change at this level. Modular innovation happens at mostly at micro architectural levels where the interfaces remain intact, but the core concepts change. I will discuss an example of radical innovation in next section about adoption of new technology and here I will be discussing how does this framework help in a case of modular innovation at micro-architectural level.

This framework helps in the innovation process in three ways:

- It helps in studying the compatibility between various options.
- It helps in devising interfaces between various systems with various options.
- It helps in evaluating the sets of various options.

We will go through an example to illustrate this. We have already discussed the micro-architecture of a motorcycle fuel cap. Now we will illustrate how this architectural framework was used for innovation to solve a grave performance issue, the leakage of evaporative emissions. With the onset of new environmental norms, Indian emission regulations put very strict limit on evaporative norms and the design illustrated in the example showed some leakages of evaporative emissions to the atmosphere. There was a thought to change the architecture to eliminate the possibility of leakage altogether. The reason, why this is an apt example to show the usefulness of this framework is that the steps used in the process can very effectively use the house of architecture. The process uses the followings steps.

- Exploring the design options: Here, the various possible options for each system are explored through brainstorming. In this case the fuel cap has three systems and the options for each of them have been shown in Table 10.
- Interface options: Here the various options for interfaces between systems are explored and are plotted in the house of architecture. The various options for interfaces are shown in Table 11.

**Table 10:** Design options for components of fuel-cap.

System A:	Cap	
Option	Detail	Illustration
a_1_	Push type cap where the evaporative gases enter and exit inside the tank itself with sealing to avoid leakage to atmosphere.	
a_2_	Push type cap where the evaporative gases do not enter the cap at all with sealing to avoid leakage to atmosphere.	
a_3_	Rotating type simple cap without lock (lock pto be provided separately).	

System B:	Metal fuel filler	
Option	Detail	Illustration
b_1_	With cam recess for cap lock and tube connecting to cap and a tube to connector	
b_2_	Internal HC collector	
b_3_	Thread matching with cap and internal HC collector	

System C:	Seal breather	
Option	Detail	Illustration
a_1_	Rubbr seal with screw holes and a connecting hole for canister tube

**Table 11:** Design options for interfaces.

Interface AB:	Cap and metal fuel filler
Option	Detail
ab_1_	Cap hinged to metal fuel filler hinged Joint
ab_2_	Cap not hinged to metal fuel filler hinged Joint
ab_3_	Thread joint

Interface BC:	Metal fuel filler and seal breather
Option	Detail
bc_1_	Seal screwed to metal fuel filler with hole aligned to canister tube
bc_2_	Seal pressing against metal fuel filler with hole aligned to canister tube
bc_3_	No seal

Interface AC:	Seal and cap
Option	Detail
ac_1_	Seal fitted with cap with hole aligned to MFF and canister tube
ac_2_	Seal pressing against cap with hole aligned to MFF and canister tube

Now, the interfaces are plotted in the house of architecture along with the coupling characteristics as shown in Table 12.

(c) Feasibility: The house of architecture is expanded to sort the feasible combinations as shown in Table 13.
(d) Evaluation: Having zeroed down to all feasible combinations, we evaluate these combinations to see the degree to which all these architectural options fulfil the functions. The degree of fulfillment of a function depends on two criterions
  - The possibility of failure
  - The level of performance

**Table 12:**
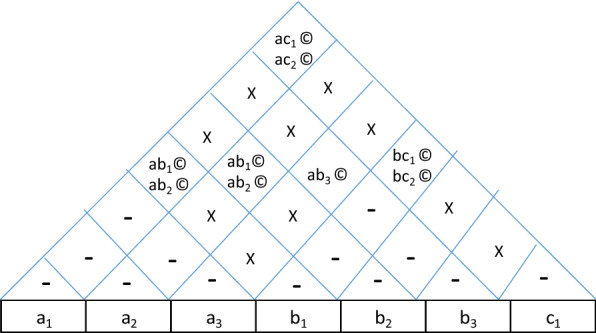
Plotting the interfaces in the house of architecture.

**Table 13:**
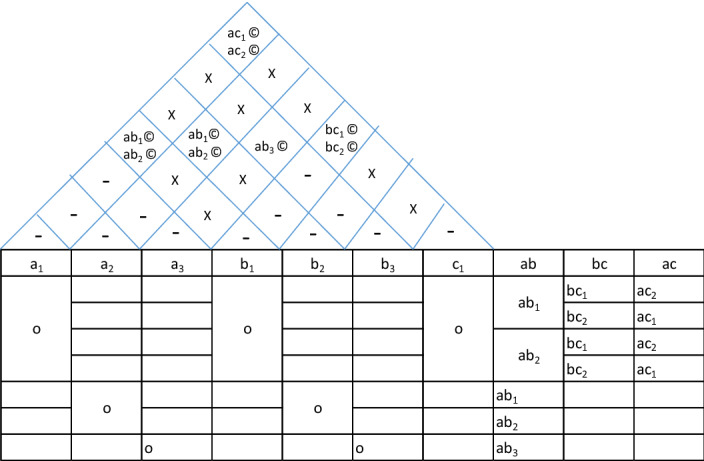
Feasibility study.

Both these criterions can be evaluated on a scale of 0 to 3 each. For the criterion of failure possibility, the rating of 0 means absolute certainty of failure, 1 means a possibility of failure if any of the parameters is at extremity of allowed variation or tolerance, 2 means possibility of failure when parameters are out of tolerance and 3 means absolutely no chances of failure. Similarly, for the criterion of performance level, 0 rating means inability to perform, 1 means the bare minimum level of performance, 2 means varying levels of performance and 3 means the performance at peak levels under all conditions. So, the overall scale for f(x) is 0 to 9 resulting from multiplication of the two ratings. We can see the expanded house of architecture in Table 14.

**Table 14:**
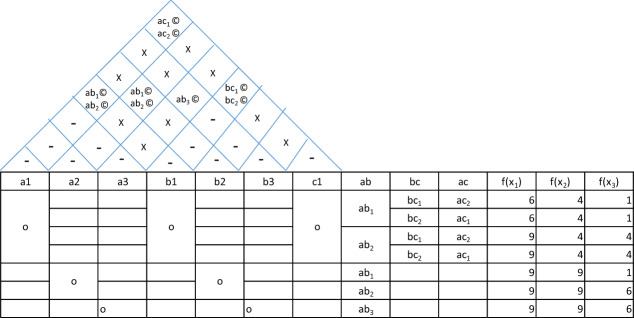
Evaluation.

**Figure 11:**
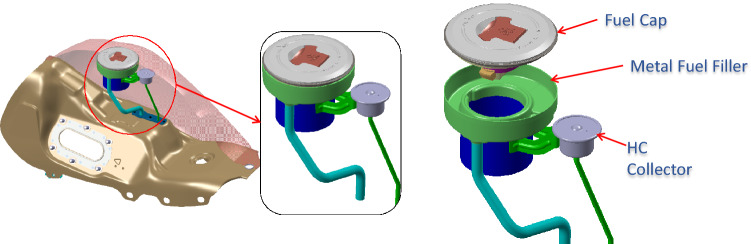
Final solution for fuel cap.

Here, it is clear from the diagram that the last two combinations meet the functions in best possible manner. But the last combination of cap with threaded locking is applicable where the cap is not an aesthetic element. In situations where cap has aesthetic value, the combination of a2, b2 without hinge gives the best possible functioning and stops the possibility of leakage altogether (ref Fig. 11).

### Adoption of new technology

Adopting new technologies for current products is a challenge and the challenge lies in making the new technology work with bare minimum changes (i.e., maintaining maximum commonality) and still maximize the benefits of new technology. Where this new architectural framework helps in achieving this is to formulate the new allocation of systems to functions and scan the network of interfaces to create and conceive new interfaces.

Here we will study the adoption of electric mobility for motorcycles. Here the benefits that are sought to be derived are cost effectiveness, non-polluting mobility with less moving parts, less frictional losses, and less noise. Here, we will start with the architecture of motorcycle we have already discussed earlier. The steps to be followed to arrive at the new architecture are as follows:

- Identify the base product and its architecture: Having already picked the base product, we start with the function-system allocation matrix (ref Table 15).
- Remove the functions which are not necessary in view of the new technology and add new functions which will be needed. Accordingly, the systems allocated to the removed functions will also be removed. Consequently, allot new systems to newly added functions. For convenience, we keep the functions and systems not affected by adoption of new technology, out of discussion and hence we remove them as well. This results in a new matrix (ref Table 16).
- For each function, explore all technological options. It is important to understand that it is the function which must be fulfilled and other considerations like construction, technology, location, sequence are all subject to change if the requirement demands it. For example, in the following table, the function of transmission system is to deliver required torque and acceleration at all speeds, and this can be fulfilled through an electrical device called controller. Similarly, with the new technology we acquire the freedom of locating the motor anywhere and therefore, in-wheel hub-motors becomes a viable option. This analysis results in the Table 17.
- Map the interfaces for all possible technological options. It is quite possible that there are two or more interfaces possible between systems. Map all of them with the conditions explicitly stated. For example, the interface between motor and rear wheel depends on whether a mechanical transmission is adopted or a controller. Moreover, there may be situations where there is no interfacing part but a relationship dictating whether the interface is coupled or decoupled. For example, the interface between brake and rear wheel is the relationship between drum and brake shoe diameters. After mapping the interfaces, we get the diagram as shown in Table 18.
- List the possible combination of options with their interfaces. For convenience and avoidance of data-clutter, we remove the technological options and their interfaces which remain the same in all options and thus do not add value to the evaluation of options, which follows the listing (ref Table 19).
- Having listed all viable combinations with their interfaces, it becomes easy to evaluate on criterions critical for selection.
- Number of mechanical and electronic systems and interfaces: Since it is simple numbers, it is easy to compare as mechanical systems increase noise and friction and therefore a smaller number of mechanical systems means smoother, less vibrating, quieter and energy efficient systems.
- Minimization of friction, noise and wear and maximization of power density: Here the rating can be comparative as exact quantification is not possible at this stage.

**Table 15:** Function-system allocation for base model.

Function	Engine	Transmission	Front wheel	Rear wheel	Fr Suspension	Rear Suspension	Fr Brake	Rear Brake	Front Frame	Frame	Front Face	Rear Body	Side Body
	1 Cyl OHC	4 speed gear transmission	Spoke and rim	Spoke and rim	Telescopic	Twin swingarm	Mechanical Drum	Mechanical Drum	Single pipe handle bar	Down Tube	Rectangular Head Light with fairing	Non-flush separate winker	3Box
Power Generation	O												
Transmission		O											
Main Frame										O			
Shock absorption					O	O							
Braking							O	O					
Traction			O										
Navigation									O				
Styling											O	O	O

**Table 16:** Function-system allocation for the new product.

	Battery	Motor	Transmission	Rear wheel	Rear brake	Frame
Power source	O					
Drive		O				
Torque Regulation			O			
Traction				O		
Braking					O	
Main frame						O

**Table 17:** System-wise design options for the new product.

Electric 2-wheeler design options			
Option	Option name	Detail	Illustration
**A: Battery: Storage, supply and renewal of energy**			
a	Battery	Liquid state Lithium Ion battery. Remaining options like fuel cell and solid state battery are not in a stage where they can be adopted for two wheelers. Moreover various options within liquid state Li-ion battery are same in architecture and do not affect the interfaces	
**B: Drive: Conversion of of stored energy into rotational energy**			
b_1_	Rigidly fixed motor	Brushless DC motor	
b_2_	In-wheel hub motor	Internal/external/axial rotation	
**C: Transmission: Regulating desired torque and speed relationship and coupling and decoupling with Output**			
c_1_	Rigid Geared transmission	Gear pairs coupled with clutch	
c_2_	In- wheel geared transmission	Axial or parallel coupling with in-wheel motor	
c_3_	Variometric transmission	Centrifugal pullys with toothed belts and centrifugal clutch	
c_4_	Controller	Torque regulation through electrical signals and decoupling after braking	
**D: Rear wheel: Traction**			
d_1_	Rear wheel	Cast or spoke	
**E: Rear brake**			
e_1_	Rear brake	Disc or drum, hydraulic or mechanical	
**F: Frame: Holding all systems**			
f_1_	Frame	Down tube, monocoque, diamond or any other conventional frame

**Table 18:**
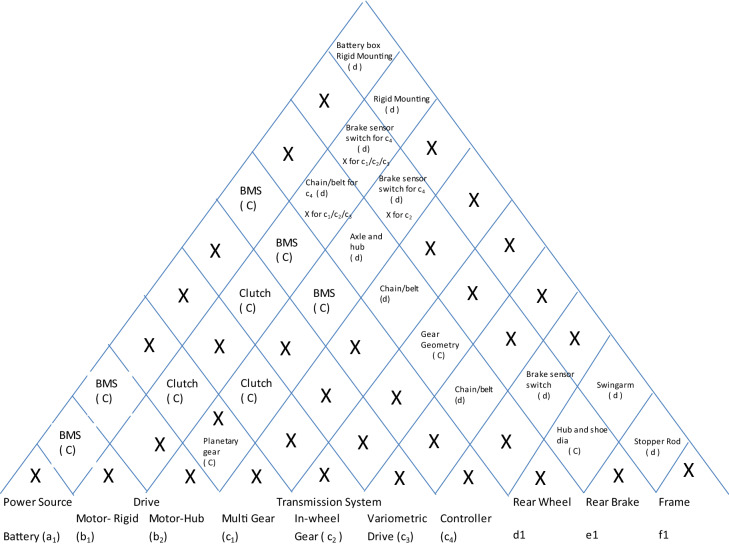
Mapping the interfaces for the new product.

**Table 19:** Listing the feasible combinations.

Options	Battery (a_1_)	Motor-rigid (b_1_)	Motor-hub (b_2_)	Multi gear (c_1_)	In-wheel gear (c_2_)	Variometric drive (c_3_)	Controller (c_4_)	d1	e1	f1	AB	BC	AC	AD	BD	CD	AE	BE	CE	DE	AF	BF
1	a1	b1		c_1_				d1	e1	f1	BMS	Clutch	X	X	X	Chain/ belt	X	X	X	Hub and shoe dia (0)	Battery box	Rigid Mtg(0)
2	a1	b1				c_3_		d1	e1	f1	BMS	Clutch	X	X	X	Chain/ belt	X	X	X	Hub and shoe dia (0)	Battery box	Rigid Mtg(0)
3	a1	b1					c_4_	d1	e1	f1	BMS			X	Chain/ belt	X	X	Brake sensor switch		Hub and shoe dia (0)	Battery box	Rigid Mtg(0)
4	a1		b_2_		C_2_			d1	e1	f1	BMS	Clutch	X	X	Axle and hub (0)	Gear-hub interface (0)	X	X	X	Hub and shoe dia (0)	Battery box	X
5	a2		b_3_				c_4_	d1	e1	f1	BMS			X	Axle and hub (0)	x	x	Brake sensor switch		Hub and shoe dia (0)	Battery box	X

So, this evaluation results in the following table (Ref Table 20).

**Table 20:** Evaluation.

Option	Systems		Interfaces		Friction minimization	Noise minimisation	Power density maximization	Wear minimization
	Mechanical	Electronic	Mechanical	Electronic				
1	2	1	3	1	1	1	1	1
2	2	1	3	1	1	1	1	1
3	1	2	2	2	3	3	3	3
4	2	1	1	1	2	2	2	2
5	1	2	0	2	4	4	4	4

With this table, it is easy to conclude that options 3 and 5 give us the best results. Though it looks that option 5 (in-wheel hub with controller) is undisputedly the best, but it has got limitations of size and capacity. Moreover, hub-motors generate high stresses for wheel-spokes which can be mitigated only up to a certain motor output and therefore, beyond a certain limit, option 3 is the best bet, as with rigidly fixed motors, the limitations are removed to a great extent.

### Standardization

With this architectural framework it is easy to identify coupled interfaces. After having identified them, it becomes possible in many cases to design the interfaces in such a way that they become decoupled interfaces. This is done by standardizing the interfaces.

**Figure 12:**
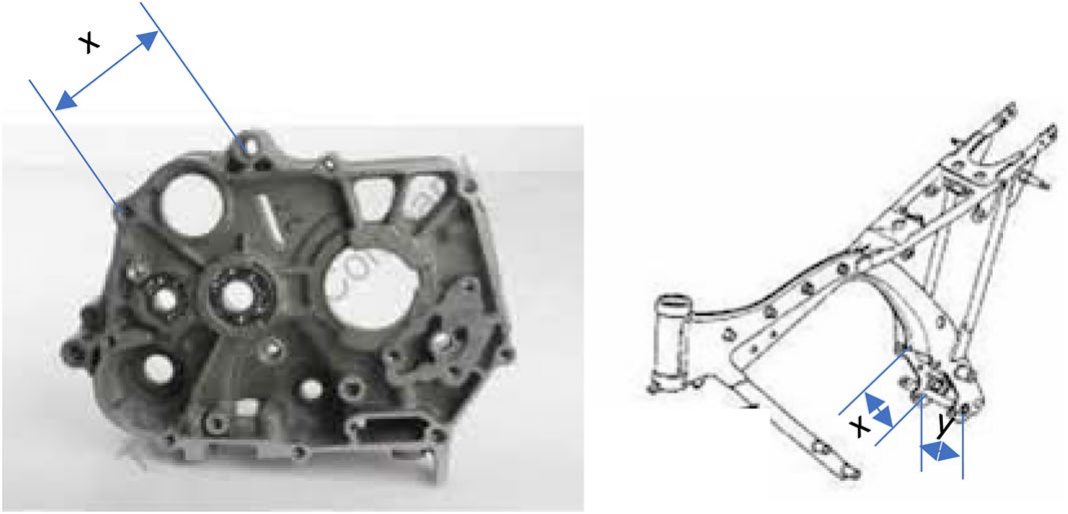
Standardizing the interfaces for engine and frame.

In the illustration shown (ref Fig. 12), the interface between engine and frame in a motorcycle is fundamentally a coupled interface as the x and y dimensions need to exactly match. To use the same engine on different frames or the same frame for different engines these dimensions need to be standardized. This requirement can be identified through the house of architecture where each interface needs to be characterized as coupled or decoupled.

## Conclusions

Referring to Chuma’ s formulation that increasing complexity of system inevitably leads to new organizational forms, this paper provides an evidence to the formulation. Various levels of components in a system increase the complexity and this has led to a new framework to organize the functions, components, interfaces, and levels. Though, Chuma’s formulation opens a completely new avenue of research to define and quantify complexity of systems and relate it to organizational forms.

The work done by Ulrich clearly establishes a definition, which is very crisp, identifiable, and verifiable. Cameron et.al and Kinnunen have provided very vivid definitions for the layers of architecture. What this paper has tried to provide is a definition for various layers (from the birds-eye to the atomic level) and their respective frameworks in the same way as house of quality provides for design quality. Though, this exercise has been completely in the domain of motorcycle design, the framework is applicable to any domain of design because the fundamental relationship between, function, systems, elements, and interfaces remains the same in all domains of design. Moreover, it has been seen that the formulations provided by Ulrich, Reich and Cameron do not suffice to conduct the processes that we have demonstrated.

The domain of innovation, as formulated by Henderson and Clark, when juxtaposed with the domain of architecture leads to a revelation about the relationship between the level of architecture and the type of innovation. Though, this paper provides an overview of this juxtaposition, this needs a new research area altogether.

To summarize, this work has added the following to the area of product architecture:

- It has identified relevant layers for automobile architecture.
- It has formulated layer-wise definitions of architecture specific to the domain of automobiles. The top layer definition relates to the interfacing between visual, engineering, and ergonomic domains whereas the system-level architecture is defined as the function-system allocation and interfacing between the systems. The component level definition is function—component allocation and the interfacing between the components at the bottom-most level. These definitions are different from those provided by Cameron et al. and Kinnunen, which are not layer specific and hence these definitions can be helpful to the product strategists and those creating computer programs at the same time.
- It has formulated layer-wise frameworks to express the architecture uniquely, crisply and in simple words. Again, these frameworks are simple to create, simple to understand and helpful in strategizing because—(a) it provides the micro and macro view at the same time and (b) function-element allocation and interfacing are uniquely described in a comprehendible way.
- After answering the basic research question of finding a level-wise framework for the definition of architecture, this paper goes on to verify whether this framework satisfies the definition of architecture which has already been established by Ulrich and finds that the answer is in affirmative.
- The next question was about the usefulness of this whole exercise and this paper has demonstrated with examples how this framework can be used for platforming strategy, innovation, adoption of new technology and standardization.

Finally, this work can be put to following applications:

- The product strategic plan for automobiles needs a simple but effective tool to express the architecture which uniquely encompasses the essential and unique aspects of automobile design. This work provides exactly that in form of top-level product architecture and house of architectures for system level, which provide a birds-eye view of the comparative architecture to the product-planners to enable them to take a decision. The models provided by Cameron et al. and Kinnunen are too complex to have a comparative birds-eye view.
- This work helps in creating a simple algorithm for capturing the architecture so that it can help in effective platform—planning, innovation, and standardization. The house of architecture is a tool that is effectively utilized for platform planning where commonization plan is derived using this tool. Similarly, the house of architecture can be very effectively used for innovation and new technology adoption the way it has been demonstrated in respective sections. Finally, for standardization, the house of architecture readily identifies the coupled interfaces.

So, this framework not only gives the designers a tool to objectively describe the architecture at various levels in automobile design but offers flexibility in all domains of design and creativity.
